# Kangri Cancer

**Published:** 1909-09-25

**Authors:** 


### Tropical Diseases.

### KANGRI CANCER.

Of all the modes in which epithelioma manifests itself there is probably none more curious nor more interesting than that peculiar form of cancer met with in Northern India, in the province of Kashmir, which is known as Kàngri Cancer. Every native of Kashmir—man, woman, and child—carries under the loose woollen robe which constitutes their only garment a wicker basket, enclosing an earthenware jar, filled with burning charcoal. Such is the primitive fashion in which these quaint people protect themselves against the intense cold which—many will be surprised to learn—is a not uncommon feature of the Kashmiri climate. In course of time the protecting wickerwork which covers the hot pot or Kàngri gets worn or charred away, and the unprotected skin of the abdomen and thighs gets more or less severely burnt, with the result that every adult Kashmiri possesses a mottled, pigmented area of scars in the region of contact with the basket.

In these areas epithelioma so frequently arises that this particular form of cancer constitutes an overwhelming majority of the total number of cases of malignant disease met with. The actual carcinoma may begin as an ulcer or as a warty growth, or may originate in an ulcer or a wart which has been in a quiescent condition for a considerable period. Sometimes one meets with a simple chronic papilloma and an undoubted epithelioma existing side by side. The growths always commence on the scarred and damaged areas which result from the burns; and when they have developed malignant characters behave exactly as do other epitheliomata arising on the skin surface under other conditions. Secondary deposits occur in the axillary or more commonly in the inguinal glands, dependent, of course, upon the site of the primary growth, *i.e.*, whether above or below the umbilicus. As to whether visceral metastases are common it is not possible to form an opinion. There is no clinical evidence pointing in that direction, but as the Kashmiri will not permit postmortem examinations there is no definite evidence.

A critical survey of a large number of cases reveals several very interesting facts about this curious condition. In the first place, although men and women must be burned and scarred with equal frequency, yet the number of cases of cancer met with in women is very small—only one quarter of the whole. This may in part be accounted for by the reluctance of the native to allow his women folk to be treated by a European doctor; but this cannot be the whole explanation, and one is forced to the conclusion that some other ætiological force is at work in addition to the presence of the precancerous areas on the skin. There is another clinical fact which points in this direction, for it it of interest to note that hardly any cases of Kàngri cancer occur before the age of forty, yet quite young children show the pigmented scars. These points fit in with the fully recognised fact that women appear to exhibit a much less liability to squamous cancer than men, and that although indubitable cases of epithelioma have been described in young children—for instance, cancer of the lip in a boy of twelve is reported—yet they are very rare before late adult life is reached.

Not the least interesting feature of Kàngri cancer is the occasional occurrence of multiple primary growths, one on each thigh, and perhaps one on the abdomen. This fact is of peculiar importance in connection with the whole question of multiple primary lesions. In the case of such widely different positions as the thigh and abdomen—especially when the peculiar circumstances of the origin of these growths are taken into consideration—there can be no reasonable hesitation in accepting them as examples of separate and distinct lesions of primary origin. And yet, in these rare cases when an epithelioma arises on each lip or two distinct ones on the same lip, the possibility of their separate origin is by many hotly denied, infection from one lip to the other or subcutaneous lymphatic spread being cited as the explanation of the phenomenon. Similarly in the case of the vulva, where multiple growths are by no means rare, the same objections have been advanced. In view of the evidence afforded by Kàngri Cancer, and also by the somewhat allied condition of *x*-ray cancer, in which, indeed, multiple tumours are the rule, it must be accepted as proved that separate and distinct carcinomata can and not infrequently do originate in the same individual.

The very marked pre-cancerous conditions associated with Kàngri cancer, and indeed with so many of the superficial cancers—*x*-ray cancer, lupus cancer, betel-nut cancer, and leukoplakia cancer—lead one to speculate as to the extent to which cancer in less accessible portions of the body, such as the breast, rectum, stomach, etc., may be similarly preceded.

